# Transmission dynamics of fractional order SVEIR model for African swine fever virus with optimal control analysis

**DOI:** 10.1038/s41598-024-78140-9

**Published:** 2024-11-08

**Authors:** S. Suganya, V. Parthiban, L. Shangerganesh, S. Hariharan

**Affiliations:** 1 Department of Mathematics, School of Advanced Sciences, Vellore Institute of Technology, Chennai Campus, Chennai, Tamilnadu 600127 India; 2 Department of Applied Sciences, National Institute of Technology Goa, Kottamoll Plateau, Cuncolim, Goa 403703 India; 3 Department of Mathematics, School of Engineering, Dayananda Sagar University, Bengaluru, 562112 India

**Keywords:** African swine fever, Caputo fractional derivative, Stability analysis, Optimal control, Numerical Simulation., Mathematics and computing, Infectious diseases

## Abstract

Understanding the dynamics of the African swine fever virus during periods of intense replication is critical for effective combatting of the rapid spread. In our research, we have developed a fractional-order SVEIR model using the Caputo derivatives to investigate this behaviour. We have established the existence and uniqueness of the solution through fixed point theory and determined the basic reproduction number using the next-generation matrix method. Our study also involves an examination of the local and global stability of disease-free equilibrium points. Additionally, we have conducted optimal control analysis with two control variables to increase the number of recovered pigs while reducing the number of those infected and exposed. We have supported our findings with numerical simulations to demonstrate the effectiveness of the control strategy.

## Introduction

Animal illnesses, particularly international animal diseases, can have substantial economic consequences at the farm, regional, and national levels due to losses in livestock output and the high costs of prevention, control, or eradication strategies. The most significant and economically destructive swine disease in existence today is without a doubt African swine fever(ASF). A member of the Asfarviridae family of DNA viruses is the cause of ASF. The first instance was noted in Kenya in 1921, where the disease is still endemic in sub-Saharan Africa. It is now widespread around the world, affecting more than 50 nations, including the Republic of Korea, China, Malaysia, Germany, Bhutan, and India. It is also present in Africa, Europe, Asia, and the Pacific, which resulted in enormous losses^1^. Due to its high mortality rate and socioeconomic impact, ASF is a notifiable illness that poses a danger to the production and commerce of pork. High fever, unconsciousness, breathing issues, vomiting, diarrhoea, appetite loss, and reddish warts near the ear, mouth, legs, and groins constitute some of the symptoms of ASF. A variety of scientific disciplines have taken part in developing efficient control strategies to reduce the spread of ASF.

In the twenty-first century, mathematical biology has become increasingly valuable to researchers. One of its primary applications is understanding and managing contagious diseases through mathematical modelling. A significant development in epidemiological research is the utilization of mathematics to grasp the dynamics of infectious disease spread^2–8^. Mathematical principles also provide insights into interactions between disease vectors and their hosts. The widespread use of these models has been facilitated by the advent of new computing technologies.

Designing real-world models, particularly those of biological systems, depends extensively on fractional calculus. Given their capacity to simulate a wide range of complicated events, fractional order differential equations (FODEs) have attracted the interest of numerous academics across a variety of disciplines, including engineering, finance, and epidemiology^9–17^. For example, adaptive synchronization, mass equations, optimal control problems, equations of motion, and chemical reactions have all been studied using FODEs recently^18,19^. Through various iterative techniques, these problems can be solved numerically. The fractional order model addresses the limitations by introducing a non-integer derivative, which allows for memory effects and long-term correlations in the system. This approach has been shown to provide a better fit to empirical data and to better capture the dynamics of infectious diseases. Therefore, the development of fractional order models represents an important advancement in epidemiological modeling. As a result, disease patterns may be described more precisely, and future outbreaks can be predicted more accurately. The effectiveness of FODEs in estimating actual data is one of its additional benefits. FODEs are frequently employed in place of traditional models because they frequently do not adequately fit the field data^20^. For instance, when compared to experimental data, the FODE model for the dengue disease outbreak was said to have done well^21^. Furthermore Caputo derivative approach is better suited for fractional modelling of epidemic diseases because it guarantees non-singular initial conditions, accounts for memory effects, provides a physical interpretation and is computationally efficient (see, for instance,^22–43^).

ASF virus has a huge negative impact on the GDP of several countries. As a result, finding a viable method to prevent the spread of this virus and control the disease is critical. Despite major scientific achievements, the World Organization for Animal Health (WOAH) highlights the importance of knowing the history and evolution of the African swine fever virus (ASFV) spread to develop strategies to reduce transmission. According to the literature, the transmission dynamics of ASF have received limited research in terms of mathematical modelling. Some studies have reported the impact of ASFV in the form of integer order derivative (see^44^). Currently, a small number of integer and non-integer systems have been established to test, investigate and comprehend the ASF virus^1,44–46^. In a relevant study, Barongo et al.^44^ present a stochastic model aimed at simulating the transmission dynamics of ASFV within a free-ranging pig population, considering different intervention scenarios.

The researchers utilized the model to evaluate the comparative impact of various prevention methods on death due to diseases. They incorporated a decay function on the transmission rate to simulate the implementation of biosecurity measures. Shi et al.^45^ introduced a basic fractional-order model to describe the transmission dynamics of African swine fever. They considered two cases: constant control and optimal control. In the former case, they established the existence and uniqueness of a positive solution, determined the basic reproduction number, and obtained sufficient conditions for the stability of two equilibria using the next-generation matrix method and Lyapunov LaSalle’s invariance principle. In the latter case, they focused on optimal control. By employing the Hamiltonian function and Pontryagin’s maximum principle, they derived the optimal control formula. Many scientists have constructed various models and conducted quantitative studies using Euler’s and Adam’s PECE methods to validate the theoretical findings. Kouidere et al.^1,46^ developed a classical and fractional model for ASFV. They conducted a sensitivity analysis of the model parameters to identify the parameters with a significant influence on the reproduction number ${\mathscr {R}}_{0}$. Based on their findings, the researchers proposed multiple strategies to effectively decrease the amount of diseased pigs and parasites.

In addition, the impact of fractional order and medical resources on system stability was investigated in^47^. The authors in^48^ developed fractional order models with media coverage, showing that timely media coverage and disinfection control measures are crucial for preventing the spread of African swine fever. For instance, the authors in^49^ analyzed a fractional order ASF model with saturation incidence, demonstrating that timely and effective disinfection measures are important to prevent disease spread. These models provide valuable insights for developing effective ASF prevention and control strategies. The vaccination strategy has recently been shown to be an effective technique for preventing disease transmission. Developing an effective vaccine for ASF has been challenging due to the complex nature of the virus. Vaccination is a widely utilized strategy of disease control. Vaccination serves as a booster to enhance and prolong the immune response, providing better protection against the disease^50,51^. The entire world has accepted the challenge of developing an ASFV vaccine. Through a successful partnership between Navetco, a Vietnamese company, and researchers from the United States Agricultural Research Institute (ARS), a momentous advancement has been realized. This joint endeavour has resulted in the development of NAVET-ASFVAC, an unprecedented vaccine designed to combat African swine fever (ASF). Notably, this vaccine stands as the world’s first commercially available solution of its kind, marking a significant milestone in the global effort to combat ASF. To model, investigate, and comprehend the ASFV, numerous mathematical models have been developed. Taking inspiration from the aforementioned studies, we created a Caputo fractional SVEIR model to investigate the effect of vaccination on ASFV transmission dynamics. To the best of our knowledge, no attempts have been made to examine the effect of vaccination on the ASFV model using the Caputo derivative. Our contributions aim to address this research gap and introduce new perspectives to the field of epidemiological modelling, providing valuable theoretical and numerical results for studying the ASFV model using Caputo derivatives. The main contributions and aspects of this paper are outlined below::

- Investigate the dynamical behavior of the Caputo fractional SVEIR model, which describes the transmission of ASF virus.
- Establish the stability analysis of the mentioned model.
- Perform the sensitivity analysis over the model parameters.
- Provide an optimal control strategy for an SVEIR model along with control interventions.

**Fig. 1:**
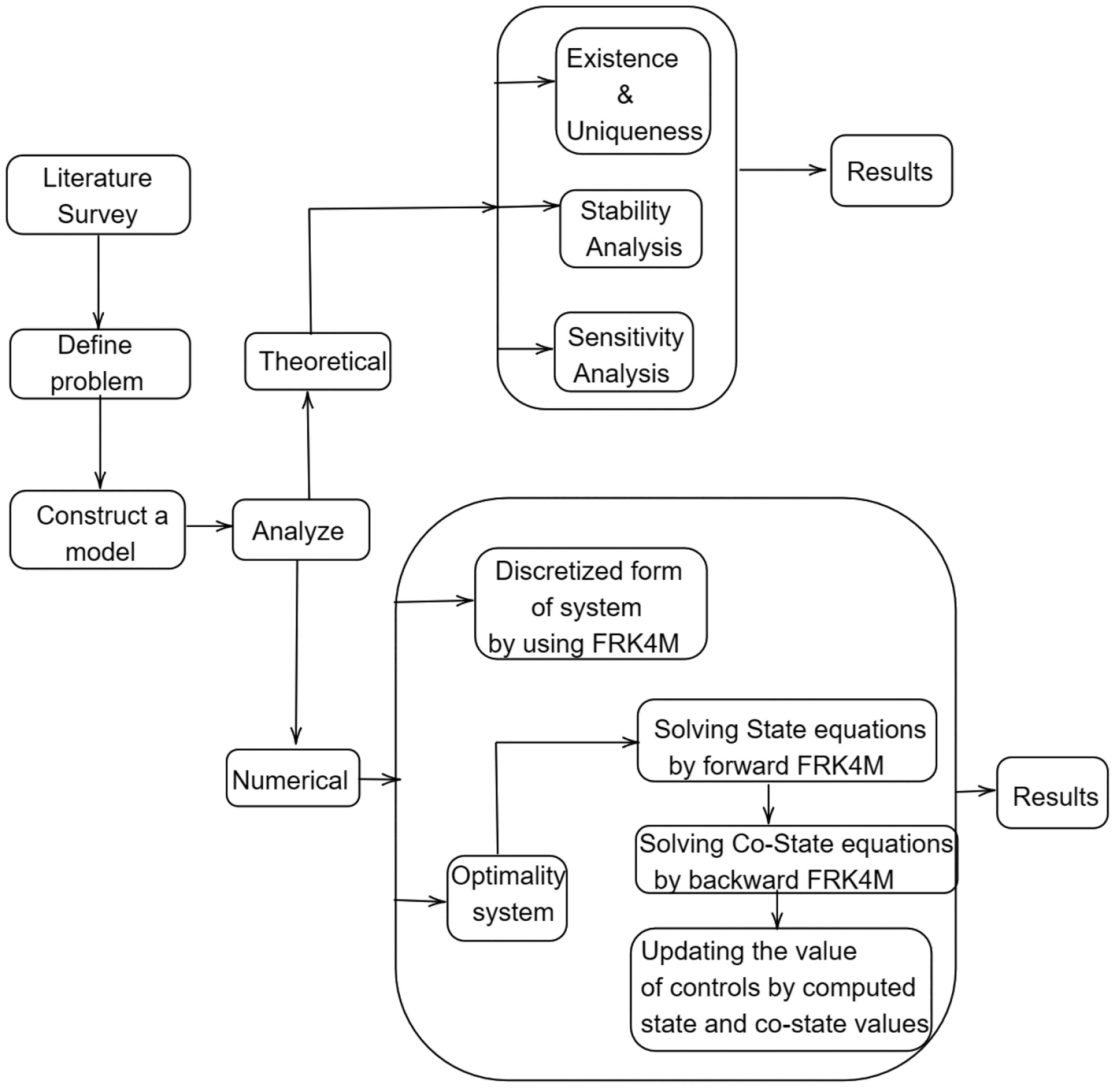
Schematic process of the proposed work.

Figure 1 depicts a schematic process of the proposed work.

In this paper, we have structured the content into several key sections. In Section “Preliminaries and methods”, we present basic concepts and preliminary studies. Moving on to Section “Formulation of the Caputo fractional SVEIR model”, we delve into the description of the Caputo fractional ASFV model. The discussion on the positivity and boundness of the system can be found in Section “Positivity and boundedness of solutions of the system”, while the exploration of existence and uniqueness results is located in Section “Existence and uniqueness results”. In Section “Equilibrium points, basic reproduction number and stability analysis of SVEIR model”, we focus on establishing the equilibria of the system and conducting a stability analysis of the basic reproduction number. Furthermore, Section “Sensitivity Analysis” contains the sensitivity analysis of the model. Section “Optimal control analysis of a SVEIR model” introduces an optimal control strategy for an SVEIR model, and the application of Pontryagin’s maximum principle is thoroughly explored in the analysis of the optimal control components. Lastly, Section “Numerical simulation results for SVEIR model” comprises numerical simulations and accompanying discussions, and the article concludes with a summary of our research.

## Preliminaries and methods

### Preliminaries

The following section of this paper addresses basic definitions and some results of the fractional-order derivatives.

#### Definition 2.1

^13^ The fractional integral of a continuous function *f*(*t*) on $L^1([0, T],{\mathbb {R}})$ of order $n-1 < \alpha \le n$ is defined as,

$$I^{\alpha } f(t) = \frac{1}{{\Gamma (\alpha )}}\int\limits_{0}^{t} {(t - s)^{{\alpha - 1}} f(s)ds,}$$

where n is a positive integer and  $\Gamma (.)$ is the Gamma function defined by   $\Gamma (\alpha )=\displaystyle \int _0^\infty {x^{\alpha -1}}{e^{-x}dx},~ \alpha >0.$

#### Definition 2.2

^13^ The Caputo fractional derivative for function $f(t)\in C^{n}$ of order $\alpha$ is defined by,

$$\begin{aligned} ^{C}D^{\alpha }f(t)=\frac{1}{\Gamma (n-\alpha )}\int _{0}^t{(t-s)^{n-\alpha -1}}{f^{n}(s)ds}, \end{aligned}$$

where $n\in N$ is such that $n-1<\alpha \le n$ and  $\Gamma (.)$ is the Gamma function defined by   $\Gamma (\alpha )= \displaystyle \int _0^\infty {x^{\alpha -1}}{e^{-x}dx},~ \alpha >0.$.

#### Definition 2.3

^13^ The Laplace transform of Caputo fractional differential operator of order ${\alpha } \in (n-1,n]$ is given by,

$$\begin{aligned} L[^{{C}}_{t_0}D^{\alpha }f(t)]=s^{\alpha }L[f(t)]-\sum _{k=0}^{n-1}s^{\alpha -k-1}f^{(k)}({t_0}). \\ \end{aligned}$$

#### Definition 2.4

^13^ The Mittag -Leffler Functions $E_{\alpha }$ and $E_{\alpha , \beta }$ defined by the power series

$$\begin{aligned} E_{\alpha }(z)=&\sum _{k=0}^{\infty } \frac{z^{k}}{\Gamma (\alpha k+1)}, \quad \alpha>0;&E_{\alpha , \beta }(z)=&\sum _{k=0}^{\infty } \frac{z^{k}}{\Gamma (\alpha k+\beta )}, \quad \alpha>0, \beta >0. \end{aligned}$$

where $\Gamma (.)$ is the Gamma function defined by   $\Gamma (\alpha )= \displaystyle \int _0^\infty {x^{\alpha -1}}{e^{-x}dx},~ \alpha >0.$

#### Lemma 2.1

^52^*Let *$x(t) \in {\mathbb {R}}^{+}$ *be a continuous function. Then, for any time* $t \ge t_0$

$$\begin{aligned} D^\alpha \left[ x(t)-x^*-x^* \ln \frac{x(t)}{x^*}\right] \le \left( 1-\frac{x^*}{x(t)}\right) D^\alpha x(t), \end{aligned}$$

$$x^* \in {\mathbb {R}}^{+}$$, *for all* $$\alpha \in (0,1).$$

#### **Theorem 2.1**

^53^(Krasnoselskii’s fixed point theorem) *Let* ${\mathbb {Y}}$ *be a non-empty set. Let* ${\textbf{C}}$ *be a closed, convex, non-empty subset of* ${\mathbb {Y}}$ *and suppose there exist two operators* ${\textbf{A}}_1, {\textbf{A}}_2$ *such that*

(i) $${\textbf{A}}_{1}\theta +{\textbf{A}}_{2}\theta \in {\textbf{C}}$$, *for all* $$\theta \in {\textbf{C}};$$
(ii) ${\textbf{A}}_{1}$ *is a contraction*;
(iii) ${\textbf{A}}_{2}$ *is compact and continuous*.

*Then there exists at least one solution* $\theta \in {\textbf{C}}$ *such that*   ${\textbf{A}}_{1}\theta + {\textbf{A}}_{2}\theta = \theta .$

## Formulation of the Caputo fractional SVEIR model

**Fig. 2:**
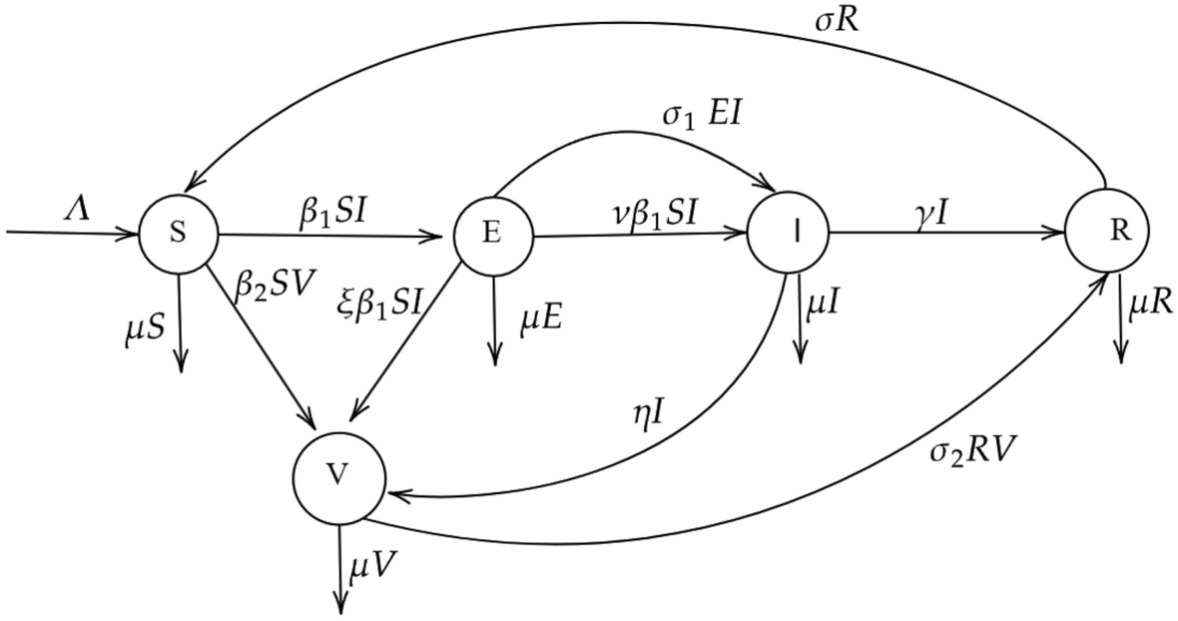
Schematic flowchart for SVEIR model.

We consider the Caputo sense nonlinear fractional order model for the African swine fever, in which the total population *N*(*t*) is assumed to comprise five compartments that include pigs susceptible *S*(*t*), the pigs vaccinated *V*(*t*), exposed pigs *E*(*t*), the pigs infected *I*(*t*) and the pigs recovered *R*(*t*), at time t $( N =S+V+E+I+R).$ The transmission dynamics of the above population are represented by the following system of nonlinear Caputo fractional order differential equations (CFODE) as

1 $$\begin{aligned} {\left\{ \begin{array}{ll} ^{C} {D}^{\alpha }S(t)=& \Lambda -\beta _{1} SI-\beta _{2} SV+\sigma R-\mu S,\\ ^{C} {D}^{\alpha }V(t)=& \beta _{2} SV+\xi \beta _{1} SI+\eta I-\sigma _{2}RV-\mu V,\\ ^{C} {D}^{\alpha } E(t)=& \beta _{1}(1-\xi -\nu ) SI-\sigma _{1}EI-\mu E,\\ ^{C} {D}^{\alpha } I(t)=& \nu \beta _{1} SI+\sigma _{1} EI-(\mu +\gamma )I- \eta I,\\ ^{C} {D}^{\alpha } R(t)=& \gamma I- (\mu +\sigma )R+\sigma _{2}RV. \end{array}\right. } \end{aligned}$$

The initial states are all positive. In this model, the parameter $\Lambda$ represents the influx of pigs. The infection rate is represented by $\beta _1$. Additionally, the immediate vaccination rate for infected pigs transitioning from the susceptible class is given by $\xi \beta _1$. The rate at which infected pigs are capable of spreading the infection to others is denoted as $\nu \beta _1$. Meanwhile, the infected pigs that do not transmit the infection to others are categorized as part of the exposed class, with a transfer rate of $\beta _1(1-\xi -\nu )$, where $1-\xi -\nu$ signifies a positive transfer rate.

The rate of administering an effective precautionary dose against infection is denoted as $\beta _2$. Some individuals recover either naturally or without the need for vaccination, represented by the rate $\gamma$. The possibility of recovering without prior infection introduces the potential for subsequent infections, expressed by the parameter $\sigma$. Vaccination administered to infected pigs is considered under the rate $\eta$. The transition of pigs to the recovered compartment due to the effectiveness of vaccination after a certain period is indicated by $\sigma _2$. The transmission from exposed pigs to infected pigs is accounted for in the proportion of $\sigma _1$. The parameter $\mu$ captures the natural death proportion of pigs. Here all the model parameter values are assumed to be positive constants. Further, the flowchart of the SVEIR model is shown in Fig. 2.

## Positivity and boundedness of solutions of the system

To be biologically well-posed, the fractional-order model solution must be positive and bounded at all periods.

### Theorem 4.1

*In Caputo system* (1), *the variables have a positive value for every t* $\ge 0$* and* $\Gamma$* is positively invariant where* $\Gamma = \{(S, V, E, I, R)\in {\mathbb {R}}^{5}_{+}:0\le N \le \displaystyle \frac{\Lambda }{\mu }\}$.

### *Proof*

First, we show that *S*(*t*), *V*(*t*), *E*(*t*), *I*(*t*) and *R*(*t*) of (1) are always positive for every t$\ge 0$. Using the system (1), that we have,

2 $$\begin{aligned} {\left\{ \begin{array}{ll} ^{C} {D}^{\alpha }S(t)_{S=0}=& \Lambda +\sigma R \ge 0,\\ ^{C} {D}^{\alpha }V(t)_{V=0}=& \xi \beta _{1} SI+\eta I\ge 0,\\ ^{C} {D}^{\alpha }E(t)_{E=0}=& \beta _{1}(1-\xi -\nu ) SI\ge 0,\\ ^{C} {D}^{\alpha }I(t)_{I=0}=& 0,\\ ^{C} {D}^{\alpha }R(t)_{R=0}=& \gamma I\ge 0.\\ \end{array}\right. } \end{aligned}$$

From the above, and by using the Generalized mean-values theorem, the positivity of the model (1) is obvious. Next from the total population of the model (1), we get,

$$\begin{aligned} ^{C} {D}^{\alpha }N(t) = \Lambda -\mu N(t), \end{aligned}$$

it indicates that

$$\begin{aligned} ^{C} {D}^{\alpha }N(t)+\mu N =\Lambda . \end{aligned}$$

Now taking the Laplace and inverse Laplace transform on both side, we obtain,

$$\begin{aligned} N(t)\le N(0){\mathbb {E}}_{\alpha ,1}(-\mu t^\alpha )+ \Lambda t^{\alpha }{\mathbb {E}}_{\alpha ,(\alpha +1)}(-\mu t^\alpha ). \end{aligned}$$

According to the Mittag Leffler function:

$$\begin{aligned} {\mathbb {E}}_\alpha ,_\beta ({\textbf{z}})={\textbf{z}}{\mathbb {E}}_{\alpha ,_\alpha +\beta }({\textbf{z}})+\frac{1}{\Gamma (\beta )}; ~~~\alpha ,\beta \ge 0. \end{aligned}$$

Hence

$$\begin{aligned} N(t)\le \Big [N(0)-\frac{\Lambda }{\mu }\Big ]{\mathbb {E}}_{\alpha ,1}(-\mu t^\alpha )+\frac{\Lambda }{\mu }. \end{aligned}$$

Thus $\lim \limits _{t\rightarrow \infty }sup~ N(t)\le \displaystyle \frac{\Lambda }{\mu },$ and hence the mentioned system (1) is bounded above by $\displaystyle \frac{\Lambda }{\mu }.$ Finally we conclude that initial states are all positive functions implies the solution space $\Gamma$ is positively invariant. $\square$

## Existence and uniqueness results

In the section, we determine the existence and uniqueness of the considered model (1) under the Caputo fractional derivative with the help of fixed point theory. The model (1) can be written as follows

3 $$\begin{aligned} \begin{array}{c} ^{C} {D}^{\alpha }S(t)={\mathbb {A}}_{1}(t,S, V, E, I, R);~~~~ ^{C} {D}^{\alpha }V(t)={\mathbb {A}}_{2}(t,S, V, E, I, R);\\ ^{C} {D}^{\alpha } E(t)={\mathbb {A}}_{3}(t,S, V, E, I, R);~~~~ ^{C} {D}^{\alpha } I(t)={\mathbb {A}}_{4}(t,S, V, E, I, R);\\ ^{C} {D}^{\alpha } R(t)={\mathbb {A}}_{5}(t,S, V, E, I, R). \end{array} \end{aligned}$$

where

4 $$\begin{aligned} {\left\{ \begin{array}{ll} {\mathbb {A}}_{1}(t,S, V, E, I, R)=& \Lambda -\beta _{1} SI-\beta _{2} SV+\sigma R-\mu S, \\ {\mathbb {A}}_{2}(t,S, V, E, I, R)= & \beta _{2} SV+\xi \beta _{1} SI+\eta I-\sigma _{2}RV-\mu V,\\ {\mathbb {A}}_{3}(t,S, V, E, I, R)=& \beta _{1}(1-\xi -\nu ) SI-\sigma _{1}EI-\mu E,\\ {\mathbb {A}}_{4}(t,S, V, E, I, R)=& \nu \beta _{1} SI+\sigma _{1} EI-(\mu +\gamma )I- \eta I,\\ {\mathbb {A}}_{5}(t,S, V, E, I, R)=& \gamma I- (\mu +\sigma )R+\sigma _{2}RV. \end{array}\right. } \end{aligned}$$

Thus the Caputo model (1) takes the form

5 $$\begin{aligned} {\left\{ \begin{array}{ll} ^{C} {D}^{\alpha }{\omega }(t)=& {\mathscr {H}}(t,{\omega }(t)), ~ t\in J,\\ {\omega }(0)=& {\omega }_{0}. \end{array}\right. } \end{aligned}$$

if

6 $$\begin{aligned} {\left\{ \begin{array}{ll} {\omega (t)}=& (S, V, E, I, R)^{'},\\ {\omega (0)}=& (S_{0},V_{0},E_{0},I_{0},R_{0})^{'},\\ {{\mathscr {H}}(t,{\omega }(t))}=& ({\mathbb {A}}_{i}(t, S, V, E, I, R)^{'}), ~~~(i=1,\cdots ,5). \end{array}\right. } \end{aligned}$$

Here $(\cdot )^{'}$ represents the transpose operation. Now we can write (5) as by the fractional integral representation,

7 $$\begin{aligned} {\left\{ \begin{array}{ll} {\omega }(t)=& {\omega }_{0}+{\mathscr {J}}_{0}^{\alpha }{{\mathscr {H}}(t,{\omega }(t)},\\ {\omega }(t)=& {\omega }_{0}+\displaystyle \frac{1}{\Gamma (\alpha )}\displaystyle \int _{0}^t{(t-\varrho )^{\alpha -1}}{{\mathscr {H}}(\varrho ,{\omega }(\varrho ))d\varrho }. \end{array}\right. } \end{aligned}$$

Let ${\mathbb {S}}$= C([0,b];${\mathbb {R}}$) be the Banach space of all continuous function from [0,b] to ${\mathbb {R}}$ provided with the norm defined by $\left\| \omega \right\| =\sup \limits _{t\in {\mathbb {J}}}|\omega (t)|$, where $|\omega (t)|=|S(t)|+|V(t)|+|E(t)|+|I(t)|+|R(t)|$ and $S, V, E, I, R\in C([0,b]).$ Suppose that ${\mathscr {H}}\in C([{\mathbb {J}},{\mathbb {R}}])$and ${\mathscr {H}}:{\mathbb {J}}\times {\mathbb {R}}^5 \rightarrow {\mathbb {R}}$ is to be continuous and bounded in order to determine existence and uniqueness of solutions. Therefore, we assume that

- $({\mathbb {A}}1)$  There exists a constants $\Phi \in C\left( [0, b], {\mathbb {R}}_{+}\right) >0$, such that$|{\mathscr {H}}(t, \omega )| \le \Phi (t),$ for all $(t,\omega )\in {\mathbb {J}}\times {\mathbb {R}}^5$.
- $({\mathbb {A}}2)$  There exists a constants ${\mathscr {L}}_{{\mathscr {H}}}>0$, $\forall t\in J$ and each ${\omega _{1}(t)},{\omega _{2}(t)}\in C$, such that
  $$\begin{aligned} |{\mathscr {H}}(t,\omega _{1})- {\mathscr {H}}(t, \omega _{2})|\le {\mathscr {L}}_{{\mathscr {H}}}|{\omega _{1}}-{\omega _{2}}|. \end{aligned}$$

By well-known Krasnoselskii’s fixed point theorem, to establish that a solutions of the system (7) exists, which is equal with the suggested model (1).

### Theorem 5.1

*Given the assumption* $({\mathbb {A}}1)$ *together with the continuity of* ${\mathscr {H}}$* then* (7)* which is equivalent with the mentioned system* (1)* has atleast one solution when* ${\mathscr {L}}_{{\mathscr {H}}}\left\| \omega _{1}\left( t_{0}\right) -\omega _{2}\left( t_{0}\right) \right\| <1.$

### *Proof*

Now Let $\sup \limits _{t \in J}|\Phi (t)|=\Vert \Phi \Vert$ and $\eta \ge \Vert \omega _{0}\Vert +\Theta \Vert \Phi \Vert ,$ and we consider ${\textbf{C}}_{\eta }=\{\omega \in {\mathbb {E}}:\Vert \omega \Vert \le \eta \}$. Let us take two operators ${\textbf{P}}_1, {\textbf{P}}_2$ on ${\textbf{C}}_{\eta }$ defined by,

$$\left( {\textbf{P}}_{1} \omega \right) (t)=\frac{1}{\Gamma (\alpha )} \int _{0}^{t}(t-\varrho )^{\alpha -1}{\mathscr {H}}(\varrho ,\omega (\varrho ))d\varrho , \quad t \in J,$$

and

$$\left( {\textbf{P}}_{2}\omega \right) (t)=\omega \left( t_{0}\right) ,\quad t\in J.$$

Thus, for any $\omega _{1},\omega _{2} \in {\textbf{C}}_{\eta }$, yields

$$\begin{aligned} \left\| \left( {\textbf{P}}_{1} \omega _{1}\right) (t)+\left( {\textbf{P}}_{2} \omega _{2}\right) (t)\right\|&\le \left\| \omega _{0}\right\| +\frac{1}{\Gamma (\alpha )}\int _{0}^{t}(t-\varrho )^{\alpha -1}\Vert {\mathscr {H}}(\varrho ,\omega _1(\varrho ))\Vert d\varrho , \\&\le \left\| \omega _{0}\right\| +\Theta \Vert \Phi \Vert , \\&\le \eta <\infty . \end{aligned}$$

Hence ${\textbf{P}}_1\omega _{1}+{\textbf{P}}_2\omega _{2}\in {\textbf{C}}_{\eta }.$ To show that ${\textbf{P}}_2$ is contraction operator. For any $\omega _{1}, \omega _{2}\in {\textbf{C}}_{\eta }$ we obtains

8 $$\begin{aligned} \left\| \left( {\textbf{P}}_{1}\omega _{1}\right) (t)- \left( {\textbf{P}}_{2}\omega _{2}\right) (t)\right\| \le \left\| \omega _{1}(t_0)-\omega _{2}(t_0)\right\| . \end{aligned}$$

The operator ${\textbf{P}}_{1}$ must also be continuous because the function ${\mathscr {H}}$ is continuous. Moreover any $t\in J$ and $\omega _{1}\in {\textbf{C}}_{\eta },$

9 $$\begin{aligned} \left\| {\textbf{P}}_{1}\omega \right\| \le \Theta \left\| \Phi \right\| <+\infty . \end{aligned}$$

Hence ${\textbf{P}}_{1}$ is uniformly bounded. Next, we need to prove the operator ${\textbf{P}}_{1}$ is compact. Let $\sup \limits _{(t,\omega )\in J\times {\textbf{C}}_{\eta }}|{\mathscr {H}}(t,\omega (t) )|= {\mathscr {H}}^{*}.$

Then for any $t_1, t_2\in J$ such that $t_2\ge t_1$ gives,

$$\begin{aligned} |({\textbf{P}}_{1}{\omega })(t_2)-&({\textbf{P}}_{1}{\omega })(t_1)|\\=&\Big |\frac{1}{\Gamma (\alpha )}\int _{0}^{t_2}{(t_2-\varrho )^{\alpha -1}}{{\mathscr {H}}}(\varrho ,\omega (\varrho ))d\varrho -\frac{1}{\Gamma (\alpha )}\int _{0}^{t_1}{(t_1-\varrho )^{\alpha -1}}{{\mathscr {H}}}(\varrho ,\omega (\varrho ))d\varrho \Big |,\\ \le&\frac{{\mathscr {H}}^{*}}{\Gamma (\alpha )}\Big |\int _{0}^{t_1}[{(t_2-\varrho )^{\alpha -1}}-{(t_1-\varrho )^{\alpha -1}}]{{\mathscr {H}}}(\varrho ,\omega (\varrho ))d\varrho \\ &\hspace{5cm}+\int _{t_1}^{t_2}{(t_2-\varrho )^{\alpha -1}}{{\mathscr {H}}}(\varrho ,\omega (\varrho ))d\varrho \Big |,\\ \le&\frac{{\mathscr {H}}^{*}}{\Gamma (\alpha )}\Big (2(t_{2}-t_{1})^{\alpha }+(t_{2}^{\alpha }-t_{1}^{\alpha })\Big )\rightarrow 0, as\hspace{0.1cm}t_{2}\rightarrow t_{1}. \end{aligned}$$

Hence, ${\textbf{P}}_{1}$ is equicontinuous and also it is relatively compact on ${\textbf{C}}_{\eta }.$ By applying the Arzela Ascoli theorem, ${\textbf{P}}_{1}$ is compact on ${\textbf{C}}_{\eta }$ because it is already proved that the operator is uniformly bounded and continuous. Thus using the Krasnoselskii’s fixed point theorem model (1) posses at least one solution on ${\mathbb {J}}$. $\square$

### Theorem 5.2

*The integral Eq.* (7)* which is equivalent with the mentioned system* (1)* has a unique solution under the assumption *$({\mathbb {A}}2)$ *provided that* $\Theta {\mathscr {L}}_{{\mathscr {H}}}<1,$* where* $\Theta ={b^\alpha }[({\Gamma {\alpha +1}})^{-1}].$

### *Proof*

Consider ${\textbf{B}}:{\mathbb {S}}\rightarrow {\mathbb {S}}$ defined by

10 $$\begin{aligned} ({\textbf{B}}\omega ) (t)=&{\omega }_{0}+\frac{1}{\Gamma (\alpha )}\int _{0}^t{(t-\varrho )^{\alpha -1}}{{\mathscr {H}}(\varrho ,{\omega }(\varrho ))d\varrho }. \end{aligned}$$

The operator ${\textbf{B}}$ is obviously well defined and the only solution to the model (1)) is merely the fixed point of ${\textbf{B}}$. Let $\sup \limits _{t\in {\mathbb {J}}}\left\| {\mathscr {H}}(t,0)\right\| =\mathbf {A_1}$ & ${\mathscr {H}}\ge \left\| \omega _0\right\| +\Theta \mathbf {A_1}.$ Therefore we need to show that ${\textbf{B}}{\mathbb {L}}_{{\mathscr {H}}}\subset {\mathbb {L}}_{{\mathscr {H}}}.$

Here ${\mathbb {L}}_{{\mathscr {H}}}=\left\{ {\omega \in {\mathbb {S}}:\left\| \omega \right\| \le {\mathscr {H}}}\right\}$ is closed and convex. Now for any $\omega \in {\mathbb {L}}_{{\mathscr {H}}},$ obtains,

$$\begin{aligned} |{{\textbf{B}}\omega }(t)|&=|{\omega }_{0}|+\frac{1}{\Gamma (\alpha )}\int _{0}^t{(t-\varrho )^{\alpha -1}}|{{\mathscr {H}}(\varrho ,{\omega }(\varrho ))d\varrho |},\\&\le {\omega }_{0}+\frac{1}{\Gamma (\alpha )}\int _{0}^t{(t-\varrho )^{\alpha -1}}{\Big [\Big |{{\mathscr {H}}(\varrho ,{\omega }(\varrho ))-{\mathscr {H}}(\varrho ,0)+{\mathscr {H}}(\varrho ,0)\Big |}\Big ]d\varrho },\\&\le {\omega }_{0}+\frac{1}{\Gamma (\alpha )}\int _{0}^t{(t-\varrho )^{\alpha -1}}{[{\mathscr {L}}_{{\mathscr {H}}}|{\omega (\varrho )|}+\mathbf {A_1}]d\varrho },\\&\le {\omega }_{0}+\frac{({\mathscr {L}}_{{\mathscr {H}}}\left\| \omega \right\| +\mathbf {A_1})}{\Gamma (\alpha )}\int _{0}^t{(t-\varrho )^{\alpha -1}}d\varrho ,\\ |{{\textbf{B}}\omega }(t)|&\le {\omega }_{0}+\frac{({\mathscr {L}}_{{\mathscr {H}}}{{\mathscr {H}}}+\mathbf {A_1})b^\alpha }{\Gamma (\alpha +1)}\le {\omega }_{0}+\Theta ({\mathscr {L}}_{{\mathscr {H}}}{{\mathscr {H}}}+\mathbf {A_1})\le {\mathscr {H}}. \end{aligned}$$

Hence the results follows, also given for any ${\omega }_{1},{\omega }_{2}\in {\mathbb {S}}$ we get

$$\begin{aligned} |({\textbf{B}}{\omega _{1}})(t)-({\textbf{B}}{\omega _{2}})(t)|=&\Big |\frac{1}{\Gamma (\alpha )}\int _{0}^t{(t-\varrho )^{\alpha -1}}[{{\mathscr {H}}}(\varrho ,{\omega _{1}}(\varrho ))-{{\mathscr {H}}}(\varrho ,{\omega _{2}}(\varrho ))]\Big |d\varrho ,\\ \le&\frac{{\mathscr {L}}_{{\mathscr {H}}}}{\Gamma (\alpha )}\int _{0}^t{(t-\varrho )^{\alpha -1}}|\omega _{1}(\varrho )-\omega _{2}(\varrho )|d\varrho ,\\ |({\textbf{B}}\omega _{1})(t)-({\textbf{B}}\omega _{2})(t)|\le&\Theta {\mathscr {L}}_{{\mathscr {H}}}|\omega _{1}(\varrho )-\omega _{2}(\varrho )|. \end{aligned}$$

As a result of the Banach contraction principle, the proposed model (1) has exactly one solution. $\square$

### Remark 5.3

By using the Krasnoselskii’s fixed point theorem and Banach tontraction principle the proposed model has a unique solution.

## Equilibrium points, basic reproduction number and stability analysis of SVEIR model

### Equilibria and their stability

In this subsection, the equilibrium points of the Caputo fractional order system (1) is derived. Depends on the model parameter, the positive real equilibrium point exists. First, the equilibrium point ${\mathbb {E}}_0$ of the system (1) is given as

$${\mathbb {E}}_{0}:=\Big [\displaystyle \frac{\Lambda }{\mu },0,0,0,0\Big ].$$

Suppose the model parameters satisfies, $R_{1}:= \Lambda \beta _{2}-\mu ^{2}>0,$ then the equilibrium point ${\mathbb {E}}_{1}$ of the system (1) exists, and is defined as

$${\mathbb {E}}_{1} :=\Big [\displaystyle \frac{\mu }{\beta _{2}},\displaystyle \frac{R_{1}}{\mu \beta _{2}},0,0,0\Big ].$$

Similarly the model parameters satisfies the conditions $R_2:=\Lambda \sigma _{2}-\mu \sigma >0$ and $R_3:=\Lambda \beta _{2}\sigma _{2}-\mu ^{2}\sigma _{2}-\mu ^{2}\beta _{2}-\mu \sigma \beta _{2}>0,$ then the another equilibrium point

$${\mathbb {E}}_{2} :=\Big [\displaystyle \frac{R_{2}}{\mu \sigma _{2}+\mu \beta _{2}},\displaystyle \frac{\mu +\sigma }{\sigma _{2}},0,0,\displaystyle \frac{R_{3}}{\sigma _{2}(\mu \sigma _{2}+\mu \beta _{2})}\Big ]$$

exists. Finally there exists a endemic equilibrium point. In this paper, the equilibrium point ${\mathbb {E}}_{2}$ is considered as disease free equilibrium(DFE) point of the system (1).

### The model basic reproduction number

The basic reproduction number (BRN), which measures the average number of secondary infections caused by the introduction of one infected person into a fully susceptible community, typically governs the dynamics and stability of a disease model. In other words, it affects whether the illness spreads over the entire population or not. It is denoted by ${\mathscr {R}}_{0}$.

To calculate the BRN (${\mathscr {R}}_{0}$) for the fractional order SVEIR model, we use the methods described in^54^. It can be obtained from the dominant eigen value of the matrix ${\mathscr {F}}{\mathscr {V}}^{-1}$ where

$$\begin{aligned} {\mathscr {F}}= \begin{pmatrix} 0 & \beta _{1}(1-\xi -\nu )S \\ 0 & \nu \beta _{1} S\\ \end{pmatrix},{\mathscr {V}} = \begin{pmatrix} \mu & 0 \\ 0 & (\mu +\gamma +\eta ) \\ \end{pmatrix}, \end{aligned}$$

Here the BRN (${\mathscr {R}}_{0}$) can be found by using realistic DFE point value ${\mathbb {E}}_{2}$. Hence we obtain the basic reproduction number (${\mathscr {R}}_{0}$) for the mentioned model (1) as follows,

$$\begin{aligned} {\mathscr {R}}_{0}= \frac{\nu \beta _{1}(\Lambda \sigma _{2}-\mu \sigma )}{(\mu +\gamma +\eta )(\mu \sigma _{2}+\mu \beta _{2})}. \end{aligned}$$

Further, we define a notation for future study,

$$\begin{aligned} {K}_{0}(S)= \frac{\nu \beta _{1}S}{(\mu +\gamma +\eta )}. \end{aligned}$$

### Local stability analysis

In this part, we delve into a detailed discussion on the local stability of the equilibrium point $({\mathbb {E}}_{0}),({\mathbb {E}}_{1})$ and $({\mathbb {E}}_{2})$ for system (1).

#### Theorem 6.1

*The equilibrium point* ${\mathbb {E}}_{0}$* is locally asymptotically stable if *${{K}_{0}}\Big (\frac{\Lambda }{\mu }\Big )< 1$* and* $R_{1}<0$* holds*.

#### *Proof*

The Jacobian matrix ${\mathbb {J}}$ of the system (1) is obtained as follows

$${\mathbb {J}}$$ =

$$\begin{aligned} \begin{pmatrix} -(\beta _{1} I+\beta _{2} V+\mu ) & -\beta _{2} S & 0 & -\beta _{1} S & \sigma \\ \beta _{2} V+\beta _{1}\xi I & (\beta _{2} S-\sigma _{2} R-\mu )& 0 & \xi \beta _{1} S+\eta & -\sigma _{2} V\\ \beta _{1}(1-\xi -\nu )I & 0 & -(\sigma _{1} I+\mu ) & \beta _{1}(1-\xi -\nu ) S-\sigma _{1}E & 0\\ \nu \beta _{1} I & 0 & \sigma _{1} I & {(\nu \beta _{1} S+\sigma _{1} E-(\mu +\gamma +\eta ))}& 0\\ 0 & \sigma _{2} R & 0 & \gamma & \sigma _{2} V-(\mu +\sigma ) \end{pmatrix}. \end{aligned}$$

The Characteristic equation of ${\mathbb {J}}({{\mathbb {E}}_{0}})$ is $\Big |{\mathbb {J}}({{\mathbb {E}}_{0}})-\lambda I\Big |$ =0 is given by

$$\begin{aligned} \left| \begin{array}{ccccc} -\mu -\lambda & -\frac{\beta _{2}\Lambda }{\mu } & 0 & -\frac{\beta _{1}\Lambda }{\mu } & \sigma \\ 0 & \frac{\beta _{2}\Lambda }{\mu }-\mu -\lambda & 0 & \frac{\xi \beta _{1}\Lambda }{\mu }+\eta & 0\\ 0 & 0 & -\mu -\lambda & \frac{\beta _{1}(1-\xi -\nu )\Lambda }{\mu } & 0\\ 0 & 0 & 0 & \frac{\nu \beta _{1}\Lambda }{\mu }-(\mu +\gamma +\eta )-\lambda & 0\\ 0 & 0 & 0 & \gamma & -(\mu +\sigma )-\lambda \end{array}\right| =0. \end{aligned}$$

Therefore, the eigenvalues are

$$\begin{aligned}\lambda _{1}=-\mu ,~~~ \lambda _{2}=-\mu ,~~~ \lambda _{3}=\frac{R_{1}}{\mu },~~~ \lambda _{4}=-\mu -\sigma ,~~~ \lambda _{5}= -\frac{\Big (\eta \mu + \gamma \mu + \mu ^{2} - \Lambda \beta _{1}\nu \Big )}{\mu }.\end{aligned}$$

Suppose the model parameters satisfies the inequality $\frac{\nu \beta _{1}\Lambda }{\mu }<(\mu +\gamma +\beta _{2})$ and $R_{1} < 0$, then all the eigen values are real negative real parts. Hence the equilibrium point at ${\mathbb {E}}_{0}$ is locally asymptotically stable. $\square$

#### Theorem 6.2

*The equilibrium point* ${\mathbb {E}}_{1}$ *is locally asymptotically stable if* ${{K}_{0}}\Big (\frac{\mu }{\beta _{2}}\Big )< 1$* with* ${R}_{1}>0$* and* ${R}_{3}<0$* holds*.

#### *Proof*

The characteristic equation of ${\mathbb {J}}({{\mathbb {E}}_{1}})$ is $\Big |{\mathbb {J}}({{\mathbb {E}}_{1}})-\lambda I\Big |$ =0 is given by

$$\begin{aligned} \left| \begin{array}{ccccc} -\mu -\frac{R_{1}}{\mu }-\lambda & -\mu & 0 & -\frac{\beta _{1}\mu }{\beta _{2}} & \sigma \\ \frac{R_{1}}{\mu }& -\lambda & 0 & \frac{\xi \beta _{1}\mu }{\beta _{2}}+\eta & \frac{-\sigma _{2}R_{1}}{\mu \beta _{2}}\\ 0 & 0 & -\mu -\lambda & \frac{\beta _{1}(1-\xi -\nu )\mu }{\beta _{2}} & 0\\ 0 & 0 & 0 & \frac{\nu \beta _{1}\mu }{\beta _{2}}-(\mu +\gamma +\eta )-\lambda & 0\\ 0 & 0 & 0 & \gamma & \frac{\sigma _{2}R_{1}}{\mu \beta _{2}}-(\mu +\sigma )-\lambda \end{array}\right| =0. \end{aligned}$$

The eigen values of ${\mathbb {J}}({{\mathbb {E}}_{1}})$ are given by the following:

$$\begin{aligned} \lambda _{1}=\lambda _{2}=-\mu ;~~\lambda _{3}=-\frac{R_{1}}{\mu };~~\lambda _{4}=\frac{-\Big (\beta _{2}\eta +\beta _{2}\gamma +\beta _{2}\mu -\beta _{1}\mu \nu \Big )}{\beta _{2}};~~\lambda _{5}=\frac{R_{1}\sigma _{2}}{\beta _{2}\mu }-(\mu +\sigma ). \end{aligned}$$

Here the eigen values are all negative real parts. Thus the equilibrium point ${\mathbb {E}}_{1}$ is locally asymptotically stable when the inequality $\displaystyle \frac{R_{1}\sigma _{2}}{\beta _{2}\mu }<(\mu +\sigma )$ and $R_{1}>0$. $\square$

#### Theorem 6.3

*The DFE point* ${\mathbb {E}}_{2}$ *is locally asymptotically stable if* ${\mathscr {R}}_{0}< 1$* and* $\min \{R_{1},R_{2},R_{3}\}>0$* holds*.

#### *Proof*

The Jacobian matrix ${\mathbb {J}}$ of the system (1) at the point ${\mathbb {E}}_{2} =\Big [\frac{R_{2}}{\mu \sigma _{2}+\mu \beta _{2}},\frac{\mu +\sigma }{\sigma _{2}},0,0,\frac{R_{3}}{\sigma _{2}(\mu \sigma _{2}+\mu \beta _{2})}\Big ]$, if $R_{2}$ and $R_{3} >0$ is given by

$$\begin{aligned} {\mathbb {J}}({{\mathbb {E}}_{2}})= \begin{pmatrix} -\mu -\frac{\beta _{2}(\mu +\sigma )}{\sigma _{2}} & -\frac{\beta _{2}R_{2}}{\mu \beta _{2}+\mu \sigma _{2}} & 0 & -\frac{\beta _{1}R_{2}}{\mu \beta _{2}+\mu \sigma _{2}} & \sigma \\ \frac{\beta _{2}(\mu +\sigma )}{\sigma _{2}}& 0& 0 & \eta +\frac{+R_{2}\beta _{1}\xi }{\mu \beta _{2}+\mu \sigma _{2}} & -\mu -\sigma \\ 0 & 0 & -\mu & \frac{\beta _{1}R_{2}(1-\xi -\nu )}{\mu \beta _{2}+\mu \sigma _{2}}& 0\\ 0 & 0 & 0 & \frac{\nu \beta _{1}R_{2}}{\mu \beta _{2}+\mu \sigma _{2}}-(\mu +\gamma +\eta ) & 0\\ 0 & \frac{R_{3}}{\mu \beta _{2}+\mu \sigma _{2}} & 0 & \gamma & 0 \end{pmatrix}. \end{aligned}$$

The characteristic equation of ${\mathbb {J}}({{\mathbb {E}}_{2}})$ is

$$(\lambda + \mu )\left( {\lambda + \frac{{\nu \beta _{1} R_{2} }}{{\mu \beta _{2} + \mu \sigma _{2} }} - \left( {\mu + \gamma + \eta } \right)} \right)\left( {\lambda ^{3} + a_{1} \lambda ^{2} + a_{2} \lambda + a_{3} } \right) = 0.$$

It can be easily seen that the two eigen values are negative, when ${\mathscr {R}}_0<1$ and the other three eigen values can be obtained from the cubic equation. Where

$$\begin{aligned} a_{1}= \mu +\frac{\beta _{2}(\mu +\sigma )}{\sigma _{2}};~~~ a_{2}=\frac{(\mu +\sigma )R_{3}}{\mu \beta _{2}+\mu \sigma _{2}};~~~ a_{3}=\frac{R_{3}\mu +R_{3}\sigma }{\sigma _{2}}. \end{aligned}$$

Hence by the Routh-Hurwitz condition the DFE point ${\mathbb {E}}_{2}$ is locally asymptotically stable if $a_{i}~(i=1,2,3)$ are positive and $a_{1}a_{2}-a_{3}>0$. Therefore we conclude that ${\mathbb {E}}_{2}$ is is locally asymptotically stable, if ${\mathscr {R}}_{0}< 1.$ $\square$

The above theorems indicate that if the model parameters satisfy the condition $K_{0}\left( \frac{\Lambda }{\mu }\right) < 1$ with $R_1 < 0$, then the system reaches the equilibrium point ${\mathbb {E}}_0$ after a certain period. However, if $R_1 > 0$, a second equilibrium point ${\mathbb {E}}_1$ emerges, which is stable when $R_3 < 0$ and the condition $K_{0}\left( \frac{\mu }{\beta _2}\right) < 1$ holds. Suppose, if $R_3 > 0$, a disease-free equilibrium point ${\mathbb {E}}_2$ exists and is locally stable when the basic reproduction number ${\mathscr {R}}_0 < 1$. This indicates that once the disease-free equilibrium point exists, it remains locally stable if ${\mathscr {R}}_0 < 1$.

### Global stability analysis

In this section, we establish the global stability of the equilibrium point $({\mathbb {E}}_{0}$ and ${\mathbb {E}}_{1})$ for the Caputo fractional model (1) by using Lyapunov and LaSalle’s invariance principle method^55^.

#### Theorem 6.4

*If* $R_{1}<0$* and* $\beta _1\Lambda -\mu ^2<0$, *then the equilibrium point* ${\mathbb {E}}_{0}$* is globally asymptotically stable*.

#### *Proof*

We consider the Lyapunov function $L=(S-S^{*})+V+E+I+R.$ Applying the Caputo derivative for the aforementioned equation and applying the lemma (2.1) we have,

$$\begin{aligned} {D}^{\alpha }L=&\left( 1-\frac{S^*}{S}\right) D^\alpha S+D^\alpha V+D^\alpha E+D^\alpha I+D^\alpha R\\ =&\left( \frac{\beta _{1}\Lambda }{\mu }-\mu \right) I+\left( \frac{\beta _{2}\Lambda }{\mu }-\mu \right) V-\frac{\Lambda ^2}{\mu S}+2{\Lambda }-\mu S-\frac{\sigma \Lambda R}{\mu S}-\mu (E+R)\\ \le&\left( \frac{\beta _{1}\Lambda }{\mu }-\mu \right) I+\left( \frac{\beta _{2}\Lambda }{\mu }-\mu \right) V-\frac{1}{\mu S}\left[ (\Lambda -\mu S)^2\right] \\ {D}^{\alpha }L\le&\left( \frac{\beta _{1}\Lambda }{\mu }-\mu \right) I+\left( \frac{\beta _{2}\Lambda }{\mu }-\mu \right) V\le 0 \text{ if } \beta _{1}\Lambda -\mu ^2<0 \text{ and } \beta _{2}\Lambda -\mu ^2<0. \end{aligned}$$

Then we obtain $^{C} {D}^{\alpha }L(t)\le 0$ for all $t\ge 0$.

According to the LaSalle’s invariance principle^55^, it is clear that ${\mathbb {E}}_{0}$ is globally asymptotically stable. $\square$

#### Theorem 6.5

*The equilibrium point *${\mathbb {E}}_{1}$ *is globally asymptotically stable if* $\beta _{1}<\beta _{2}$* and* $\sigma _{2}R_1<{\mu ^{2}}\beta _{2}.$

#### *Proof*

Let us define the Lyapunov function as $L=(S-S^{*})+(V-V^{*})+E+I+R.$ Then

$$\begin{aligned} {D}^{\alpha }L=&\left( 1-\frac{S^*}{S}\right) D^\alpha S+\left( 1-\frac{V^*}{V}\right) D^\alpha V+D^\alpha E+D^\alpha I+D^\alpha R\\ =&\Lambda -\mu (S+V+E+I+R)-\frac{\Lambda {S^*}}{S}+\beta _{1} {S^*}I+\beta _{2} {S^*}V-\frac{\sigma R {S^*}}{S}+\mu {S^*}- \beta _{2} S{V^*}\\&\hspace{5cm}-\frac{\xi \beta _{1}SI{V^*}}{V}-\frac{\eta {V^*}I}{V}+\sigma _{2}R{V^*}+\mu {V^*}\\&\le 2\Lambda -\frac{\Lambda \mu }{\beta _{2} S}-\frac{S\Lambda \beta _{2}}{\mu }-\mu E+ \left( \frac{\beta _{1}}{\beta _{2}}-1\right) \mu I+ \frac{\sigma _{2}R\Lambda }{\mu }-\frac{\sigma _{2}R\mu }{\beta _{2}}-\mu R\\&\le \left( \frac{\beta _{1}}{\beta _{2}}-1\right) \mu I+\left( \frac{\sigma _{2}\Lambda }{\mu }-\frac{\sigma _{2}\mu }{\beta _{2}}-\mu \right) R\\ {D}^{\alpha }L&\le 0 \text{ if } \beta _{1}<\beta _{2} \text{ and } \frac{\sigma _{2}}{{\mu ^{2}}\beta _{2}}<\frac{1}{R_{1}}. \end{aligned}$$

Then we obtain $^{C} {D}^{\alpha }L(t)\le 0$ for all $t\ge 0$. The invariant set of system (1) on the set $\{(S, V, E, I, R)\in \Gamma : ^{C} {D}^{\alpha }L(t) = 0\}$ is the singleton $\{{\mathbb {E}}_{1}\}$. Hence ${\mathbb {E}}_{1}$ is globally asymptotically stable. $\square$

## Sensitivity analysis

This section presents the sensitivity analysis for SVEIR Caputo model for ASFV. It is important to highlight that the impact of a parameter is most pronounced when its sensitivity index value is higher. The positive and negative signs in the analysis demonstrate the association between these parameters and the analyzed variables, specifically the basic reproduction number. Conducting a parameter sensitivity analysis will help identify the necessary measures to halt the transmission of ASFV.

The sensitivity index ${\mathscr {R}}_{0}$ with respect to the parameters are calculated using partial derivatives , and it can be obtain as follows:

$$\begin{aligned} \begin{aligned} \frac{\partial {\mathscr {R}}_{0}}{\partial \Lambda }&=\frac{\beta _1\nu \sigma _2}{(\beta _2\mu + \mu \sigma _2)(\gamma + \eta + \mu )}>0;&\frac{\partial {\mathscr {R}}_{0}}{\partial \beta _1}&=\frac{(\nu (\Lambda \sigma _2 - \mu \sigma ))}{((\beta _2\mu + \mu \sigma _2)(\gamma + \eta + \mu ))}; \\ \frac{\partial {\mathscr {R}}_{0}}{\partial \beta _2}&=-\frac{(\beta _1\mu \nu (\Lambda \sigma _2 - \mu \sigma ))}{((\beta _2\mu + \mu \sigma _2)^2(\gamma + \eta + \mu ))};&\frac{\partial {\mathscr {R}}_{0}}{\partial \nu }&=\frac{(\beta _1(\Lambda \sigma _2 - \mu \sigma ))}{((\beta _2\mu + \mu \sigma _2)(\gamma + \eta + \mu ))};\\ \frac{\partial {\mathscr {R}}_{0}}{\partial \sigma }&=-\frac{(\beta _1\mu \nu )}{((\beta _2\mu + \mu \sigma _2)(\gamma + \eta + \mu ))}<0;&\frac{\partial {\mathscr {R}}_{0}}{\partial \eta }&=-\frac{(\beta _1\nu (\Lambda \sigma _2 - \mu \sigma ))}{((\beta _2\mu + \mu \sigma _2)(\gamma + \eta + \mu )^{2})}; \\ \frac{\partial {\mathscr {R}}_{0}}{\partial \sigma _2}&=\frac{(\beta _1\nu (\Lambda \beta _2 + \mu \sigma ))}{(\mu (\beta _2 + \sigma _2)^{2}(\gamma + \eta + \mu ))}>0;&\frac{\partial {\mathscr {R}}_{0}}{\partial \gamma }&=-\frac{(\beta _1\nu (\Lambda \sigma _2 - \mu \sigma ))}{((\beta _2\mu + \mu \sigma _2)(\gamma + \eta + \mu )^{2})}. \end{aligned} \end{aligned}$$

From the analysis, it is evident that an increase in the total population leads to a rise in the number of infected individuals within the system. Conversely, recovering pigs from the susceptible compartment or administering vaccination significantly reduces the spread of infection. Furthermore, if the model parameters satisfy the condition $\Lambda \sigma _2 - \mu \sigma > 0$, an increase in the infection rate among pigs will complicate the system by facilitating further spread of the infection. Providing vaccinations to both susceptible and infected pigs is an effective strategy to mitigate the transmission of disease. From this study, we observed that controlling the vaccination rate $\eta$ and the recovery rate $\sigma$ plays a crucial role in effectively reducing the spread of infection.

## Optimal control analysis of a SVEIR model

In this section, we apply optimal control theory techniques to develop an effective strategy for limiting the transmission of ASF virus in pigs. In order to incorporate the efficiency of vaccination using a control measure for the system (1), we introduce two controls variables namely $u_{1}$ and $u_{2}$. Here the tightening bio-security measures at a given time t served as the first control, as denoted by $u_{1}(t)$ and the second control $u_{2}(t)$, represented the efficient disinfectant at *t*. However, iron fencing can be an important component of a bio-security plan to help prevent the contact and spread of the virus on pig farms. Implement a rigorous sanitation program to reduce the risk of introducing or spreading ASF. This includes regularly cleaning and disinfecting all equipment and vehicles that come into contact with pigs or their environment. These measures work together to create a comprehensive approach that helps to minimize the risk of ASF transmission and protect the health of the pigs on the farm.

Optimal control theory is a mathematical framework used to find the best control strategies for a given system, typically by optimizing an objective function. In the context of epidemic diseases, optimal control theory can be applied to model the spread of the disease and determine effective intervention strategies to mitigate its impact^56–61^. Agarwal et al.^62^ and Ding et al.^63^ have significantly enhanced the theory of optimal control within the field of fractional calculus through their valuable contributions. Pontryagain’s maximal principle (see^64^) is a cornerstone of the fundamental concept of optimal control in the realm of fractional calculus.

The system of equations is modified after the inclusion of the time-dependent control is as follows:

11 $$\begin{aligned} {\left\{ \begin{array}{ll} ^{C} {D}^{\alpha }S(t)=& \Lambda -\beta _{1} SI-\beta _{2} SV+\sigma u_{2}(t)R-\mu S,\\ ^{C} {D}^{\alpha }V(t)=& \beta _{2} SV+\xi \beta _{1} SI+\eta u_{1}(t)I-\sigma _{2}RV-\mu V,\\ ^{C} {D}^{\alpha } E(t)=& \beta _{1}(1-\xi -\nu ) SI-\sigma _{1}EI-\mu E,\\ ^{C} {D}^{\alpha } I(t)=& \nu \beta _{1} SI+\sigma _{1} EI-(\mu +\gamma )I- \eta u_{1}(t)I,\\ ^{C} {D}^{\alpha } R(t)=& \gamma I- (\mu +\sigma u_{2}(t))R+\sigma _{2}RV, \end{array}\right. } \end{aligned}$$

with the initial states *S*(0),  *V*(0),  *I*(0),  *E*(0) and *R*(0) are all non negative.

*Remark: The SVEIR model with control variables* $u_1$ *and* $u_2$ *is proposed after noticing the importance of model parameter from the sensitivity analysis of the basic reproduction number. *

These two control functions are both limited and Lebesgue integrable on [0, $T_f$], where $T_f$ is the fixed time interval length to which controls are applied.

Our goals here are to reduce the number of infected pigs by increasing recovered pigs population and reducing exposed and infected pigs, as well as to reduce the costs associated with controls. It can be quantitatively described by optimizing the cost functional:

12 $$\begin{aligned} {\textbf{J}}(u_{1},u_{2})=\int _{0}^{T_{f}}\Big [A_{1}E(t)+A_{2}I(t)+\displaystyle \frac{c_{1}}{2}u_{1}^{2}(t)+\displaystyle \frac{c_{2}}{2}u_{2}^{2}(t)\Big ] dt. \end{aligned}$$

Where $A_{1}$ and $A_{2}$ represents the positive weights and $c_{1}$ and $c_{2}$ are the measure of relative cost of the intervention strategies of the control $u_{1}$ and $u_{2}$ respectively. Objective is to find the control parameters $u_{1}^{*}$ and $u_{2}^{*}$, such that,

13 $$\begin{aligned} {\textbf{J}}(u_{1}^{*},u_{2}^{*})=\displaystyle \min _{u_{1},u_{2}\in {\textbf{U}}}{\textbf{J}}(u_{1},u_{2}). \end{aligned}$$

Here the control set ${\textbf{U}}$ is defined by,

14 $$\begin{aligned} {\textbf{U}}=\left\{ (u_{1},u_{2})/ 0 \le u_{\min } \le u_i(t) \le u_{\max } \le 1, i=\{1; 2\} / t \in [0, T_{f}] \right\} . \end{aligned}$$

### Characterization of optimal control functions

Pontryagin maximum principle is used to derive the necessary condition for optimality conditions for our system. In order to do this we define Hamiltonian ${\mathbb {H}}$ of the problem (12) at time *t* is defined by,

15 $$\begin{aligned} \begin{array}{ll} {\mathbb {H}}(t)=A_{1}E(t)+A_{2}I(t)+\displaystyle \frac{c_{1}}{2}u_{1}^{2}(t)& +\displaystyle \frac{c_{2}}{2}u_{2}^{2}(t)+\lambda _{1} ^{C} {D}^{\alpha }S(t)+\lambda _{2} ^{C} {D}^{\alpha }V(t)\\ & +\lambda _{3} ^{C} {D}^{\alpha }E(t)+\lambda _{4} ^{C} {D}^{\alpha }I(t)+\lambda _{5} ^{C} {D}^{\alpha }R(t). \end{array} \end{aligned}$$

Here $\lambda _{j}'s$ are the co-state variables for $j = 1,2\ldots 5.$ with the co-state equations as follows:

$$\begin{aligned} \begin{array}{c} {D}^{\alpha }_{T_{f}} \lambda _{1}(t)=-\displaystyle \frac{\partial {\mathbb {H}}(t)}{\partial S(t)};~~~~ {D}^{\alpha }_{T_{f}} \lambda _{2}(t)=-\displaystyle \frac{\partial {\mathbb {H}}(t)}{\partial V(t)};~~~~ {D}^{\alpha }_{T_{f}} \lambda _{3}(t)=-\displaystyle \frac{\partial {\mathbb {H}}(t)}{\partial E(t)};\\ {D}^{\alpha }_{T_{f}} \lambda _{4}(t)=-\displaystyle \frac{\partial {\mathbb {H}}(t)}{\partial I(t)};~~~~ {D}^{\alpha }_{T_{f}} \lambda _{5}(t)=-\displaystyle \frac{\partial {\mathbb {H}}(t)}{\partial R(t)}. \end{array} \end{aligned}$$

### Necessary optimality conditions

#### Theorem 8.1

*The optimal controls *$u_{1}^{*}$* and *$u_{2}^{*}$* and corresponding solutions of the state Eq.* (11) are $S^{*}, V^{*}, E^{*},I^{*}$* and* $R^{*}$ *then there exists co-state variables* $\lambda _{1},\lambda _{2},\lambda _{3},\lambda _{4}$* and* $\lambda _{5}$* satisfying the following:*

$$\begin{aligned} {D}^{\alpha }_{T_{f}} \lambda _{1}(t)=-\frac{\partial {\mathbb {H}}(t)}{\partial S(t)}=&-\lambda _{1}(t)\Big [ \beta _{1}I-\beta _{2}V-\mu \Big ]-\lambda _{2}(t)\Big [\beta _{2}V+\xi \beta _{1} I\Big ]\\ &\hspace{3cm}-\lambda _{3}(t)\Big [\beta _{1}(1-\xi -\nu ) I\Big ]-\lambda _{4}(t)\Big [\nu \beta _{1}I\Big ]\\ {D}^{\alpha }_{T_{f}} \lambda _{2}(t)=-\frac{\partial {\mathbb {H}}(t)}{\partial V(t)}=&\lambda _{1}(t)\Big [ \beta _{2}S\Big ]-\lambda _{2}(t)\Big [\beta _{2}S-\sigma _{2}R-\mu \Big ]-\lambda _{5}(t)\Big [\sigma _{2}R\Big ]\\ {D}^{\alpha }_{T_{f}} \lambda _{3}(t)=-\frac{\partial {\mathbb {H}}(t)}{\partial E(t)}=&-A_{1}+\lambda _{3}(t)\Big [\sigma _{1}I+\mu \Big ]-\lambda _{4}(t)\Big [\sigma _{1}I\Big ]\\ {D}^{\alpha }_{T_{f}} \lambda _{4}(t)=-\frac{\partial {\mathbb {H}}(t)}{\partial I(t)}=&\lambda _{1}(t)\beta _{1}S-\lambda _{2}(t)\Big [\xi \beta _{1} S+\eta u_{1}(t)\Big ]-\lambda _{3}(t)\Big [\beta _{1}(1-\xi -\nu ) S-\sigma _{1}E\Big ]\\ &\hspace{1cm}-A_{2}-\lambda _{4}(t)\Big [\nu \beta _{1}S+\sigma _{1}E-\mu -\gamma -\eta u_{1}(t)\Big ]-\lambda _{5}(t)\Big [\gamma \Big ]\\ {D}^{\alpha }_{T_{f}} \lambda _{5}(t)=-\frac{\partial {\mathbb {H}}(t)}{\partial R(t)}=&-\lambda _{1}(t)\Big [\sigma u_{2}(t)\Big ]+\lambda _{2}(t)\Big [\sigma _{2}V\Big ]+\lambda _{5}(t)\Big [\mu +\sigma u_{2}(t)-\sigma _{2}V\Big ], \end{aligned}$$

* with the transversality condition* $\lambda _{1}(T_{f})=\lambda _{2}(T_{f})=\lambda _{5}(T_{f})=0$* and *$\lambda _{3}(T_{f})=-A_{1},~\lambda _{4}(T_{f})=-A_{2}$.* Moreover the objective function J is minimized within the region U by the optimal controls* $u_{1}^{*}$* and* $u_{2}^{*}$* are given by,*

$$\begin{aligned} u_1^*(t)=&\min \left\{ \max \left\{ 0, \frac{\left( \lambda _4-\lambda _2\right) \eta I^*(t)}{c_{1}} \right\} , 1\right\} ;\\ u_2^*(t)=&\min \left\{ \max \left\{ 0, \frac{\left( \lambda _5-\lambda _1\right) \sigma R^*(t)}{c_{2}} \right\} , 1\right\} . \end{aligned}$$

#### *Proof*

Let us define the Hamiltonian function as follows:

$$\begin{aligned} {\mathbb {H}}(t)=&A_{1}E(t)+A_{2}I(t)+\frac{c_{1}}{2}u_{1}^{2}(t)+\frac{c_{2}}{2}u_{2}^{2}(t)+\lambda _{1}(t)\Big [\Lambda -\beta _{1} SI-\beta _{2} SV+\sigma u_{2}(t)R-\mu S\Big ]\\&+\lambda _{2}(t)\Big [\beta _{2} SV+\xi \beta _{1} SI+\eta u_{1}(t)I-\sigma _{2}RV-\mu V\Big ]+\lambda _{3}(t)\Big [\beta _{1}(1-\xi -\nu ) SI-\sigma _{1}EI-\mu E\Big ]\\&+\lambda _{4}(t)\Big [\nu \beta _{1} SI+\sigma _{1} EI-(\mu +\gamma )I- \eta u_{1}(t)I \Big ]+\lambda _{5}(t)\Big [\gamma I- (\mu +\sigma u_{2}(t))R+\sigma _{2}RV \Big ]. \end{aligned}$$

By employing Pontryagin’s maximum principle, we can derive the adjoint equations and transversality conditions for all t within the interval [0, $T_{f}$]. We obtain the co state equations as follows:

$$\begin{aligned} {D}^{\alpha }_{T_{f}} \lambda _{1}(t)=-\frac{\partial {\mathbb {H}}(t)}{\partial S(t)}=&-\lambda _{1}(t)\Big [ \beta _{1}I-\beta _{2}V-\mu \Big ]-\lambda _{2}(t)\Big [\beta _{2}V+\xi \beta _{1} I\Big ]\\ &\hspace{3cm}-\lambda _{3}(t)\Big [\beta _{1}(1-\xi -\nu ) I\Big ]-\lambda _{4}(t)\Big [\nu \beta _{1}I\Big ] \\ {D}^{\alpha }_{T_{f}} \lambda _{2}(t)=-\frac{\partial {\mathbb {H}}(t)}{\partial V(t)}=&\lambda _{1}(t)\Big [ \beta _{2}S\Big ]-\lambda _{2}(t)\Big [\beta _{2}S-\sigma _{2}R-\mu \Big ]-\lambda _{5}(t)\Big [\sigma _{2}R\Big ]\\ {D}^{\alpha }_{T_{f}} \lambda _{3}(t)=-\frac{\partial {\mathbb {H}}(t)}{\partial E(t)}=&-A_{1}+\lambda _{3}(t)\Big [\sigma _{1}I+\mu \Big ]-\lambda _{4}(t)\Big [\sigma _{1}I\Big ]\\ {D}^{\alpha }_{T_{f}} \lambda _{4}(t)=-\frac{\partial {\mathbb {H}}(t)}{\partial I(t)}=&\lambda _{1}(t)\beta _{1}S-\lambda _{2}(t)\Big [\xi \beta _{1} S+\eta u_{1}(t)\Big ]-\lambda _{3}(t)\Big [\beta _{1}(1-\xi -\nu ) S-\sigma _{1}E\Big ]\\ &\hspace{1cm}-A_{2}-\lambda _{4}(t)\Big [\nu \beta _{1}S+\sigma _{1}E-\mu -\gamma -\eta u_{1}(t)\Big ]-\lambda _{5}(t)\Big [\gamma \Big ]\\ {D}^{\alpha }_{T_{f}} \lambda _{5}(t)=-\frac{\partial {\mathbb {H}}(t)}{\partial R(t)}=&-\lambda _{1}(t)\Big [\sigma u_{2}(t)\Big ]+\lambda _{2}(t)\Big [\sigma _{2}V\Big ]+\lambda _{5}(t)\Big [\mu +\sigma u_{2}(t)-\sigma _{2}V\Big ] \end{aligned}$$

For $t \in [0,T_{f}]$ the transversality condition $\lambda _{1}(T_{f})=\lambda _{2}(T_{f})=\lambda _{5}(T_{f})=0,$ and $\lambda _{3}(T_{f})=-A_{1},\lambda _{4}(T_{f})=-A_{2}.$ Further using Pontryagin’s maximum principle we obtain the optimality controls $u_1^*(t)$ and $u_2^*(t)$

16 $$\begin{aligned} \frac{\partial {\mathbb {H}}(t)}{\partial u_1(t)}=0&\Rightarrow c_{1}u_1+(\lambda _{2}-\lambda _{4})(\eta I)=0;&\frac{\partial {\mathbb {H}}(t)}{\partial u_2(t)}=0&\Rightarrow c_{2}u_2+(\lambda _{1}-\lambda _{5})(\sigma R)=0. \end{aligned}$$

From the minimum of the cost functional ${\textbf{J}}$ we obtain the optimal controls $u_{1}^{*}$ and $u_{2}^{*}$ as follows

$$\begin{aligned} u_1^*(t)=&\min \left\{ \max \left\{ 0, \frac{\left( \lambda _4-\lambda _2\right) \eta I^*(t)}{c_{1}} \right\} , 1\right\} ;\\u_2^*(t)=&\min \left\{ \max \left\{ 0, \frac{\left( \lambda _5-\lambda _1\right) \sigma R^*(t)}{c_{2}} \right\} , 1\right\} . \end{aligned}$$

This completes the proof. $\square$

## Numerical simulation results for SVEIR model

This section provides an overview of the numerical methods employed in our study. In subsection (9.1), we utilized the FRK4M to solve the discretized form of Caputo system (1) through numerical computation. Furthermore, in subsection (9.2), we apply the forward backward sweep method (FBSM) using the FRK4M to solve the optimality system (11). These methods provide precise numerical solutions over extended time intervals. MATLAB software is employed for simulations, utilizing the specified initial conditions and parameters.

### Fractional Runge Kutta method of the fourth order

This FRK4M, an extension of the classical Runge-Kutta technique, is particularly adept at handling systems involving fractional differential equations^65–68^. To demonstrate the utilization of the FRK4M in solving the model presented in (1), we begin by considering the general form of the fractional differential equation (FDE),

17 $$\begin{aligned} \begin{array}{ll} {D}^{\alpha }{g}(t)=& f(t,{g}(t)), 0<\alpha \le 1, 0<t\le T,\\ {g}(0)=& {g}_{0}.\end{array} \end{aligned}$$

To formulate the numerical scheme for the FRK4M , we partition the interval [0, *T*] into *n* equal subintervals using points ${t_0, t_1, \cdots , t_n}$, where $t_0 = 0, ~t_j = jh$ for $j = 1, 2, \cdots , n,$ and $t_n = T,$ with $h =\displaystyle \frac{T}{n}$ representing the step size. The formulation of the FRK4M numerical scheme is represented as follows:

$$\begin{aligned} K_1=h^*f\left( t_n, g_n\right) ,~~ K_2=h^*f\left( t_n+\frac{h}{2}, g_n+\frac{K_1}{2} \right) ,&~~ K_3=h^*f\left( t_n+\frac{h}{2} , g_n+\frac{K_2}{2}\right) ,\\ K_4=h^*f\left( t_n+h, g_n+K_3\right) , ~~~~g_{n+1}=g_n+&\frac{1}{6}\left( K_1+2 K_2+2 K_3+K_4\right) , \quad \text{ where } h^*=\frac{h^\alpha }{\Gamma (\alpha +1)}.&\end{aligned}$$

Now, we observe the approximated solutions for *S*(*t*), *V*(*t*), *E*(*t*), *I*(*t*) and *R*(*t*) utilizing a step size of 0.01 for various values of the fractional order $0 <\alpha \le 1.$ The initial values are $S(0) = 1.5, V(0) = 0.3, E(0) = 0.8, I(0) = 1,$ and $R(0) = 0.2$ along with the parameter values utilized are as specified in Table 1.

**Table 1:** Model parameter values.

Parameter	$\Lambda$	$\beta _1$	$\beta _2$	$\sigma$	$\mu$	$\xi$	$\eta$	$\sigma _1$	$\sigma _2$	$\nu$	$\gamma$
Value	1	0.26	0.2	0.9	0.09	0.2	0.2	0.1	0.3	0.4	0.1

We performed numerical simulations with initial values and specified parameters as in Table 1 for the system (1) using both classical and a range of fractional order values, encompassing $\alpha = 0.75, 0.85$, and 1.

**Fig. 3:**
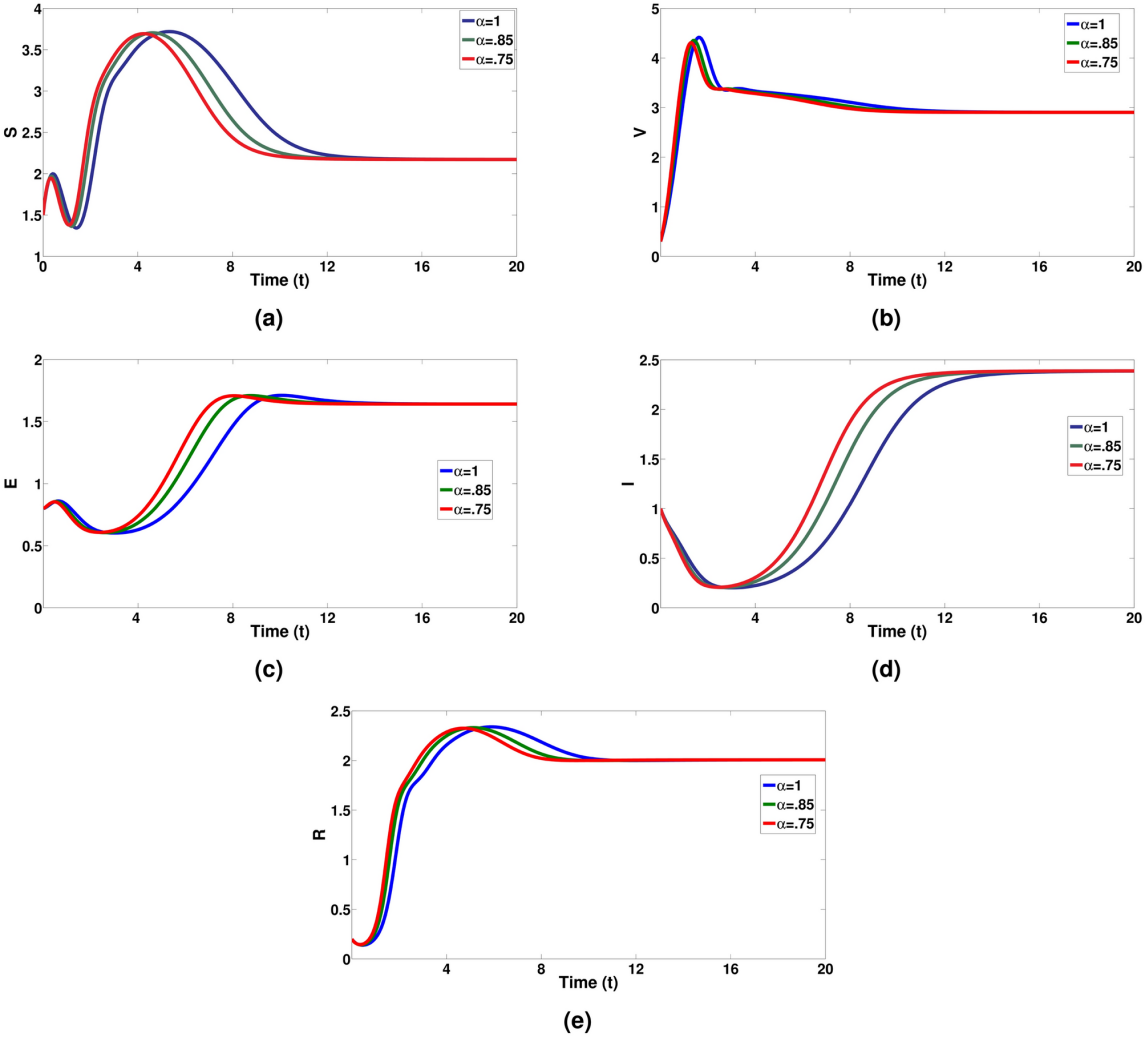
Visualizes the dynamical behaviour of all pigs population with respect to days for a integer and non-integer values of $\alpha = 0.75 \& 0.85$.

It’s great to see the different dynamics represented in the figures. The visual depiction in Fig. 3 effectively showcases how the fractional disease model provides a deeper understanding of the disease behaviour. In Fig. 3a, the evolution of susceptible pigs over time, varying both classical and different fractional orders of $\alpha$, is presented, demonstrating the impact of varying fractional orders of $\alpha$ on the proportion of susceptible pigs. Figure 3b illustrates the changes in vaccinated pigs over time, showing how they increase and then slowly decline due to the $\alpha$ values. Figure 3c–e depict the dynamical behaviour of exposed pigs over time, confirming a significant increase in the proportion of exposed, infected, and recovered pigs.

### FBSM using FRK4M

FBSM represents a highly efficient iterative method for addressing optimality systems. Building upon the foundation of FRK4M, we have enhanced FBSM to tackle our FOCP. The procedure commences with an initial estimation of the control variable. Subsequently, the state equations are solved forward in time simultaneously, while the adjoint equations are solved backwards in time. The control variable is updated using the newly computed state and adjoint values, and this iterative process continues until convergence is achieved.

Next, we discuss the numerical simulations of FOCP of the mentioned system (1). We have acquired the solutions of the optimality system using the algorithm discussed, employing the state variables with in the initial values as $S(0) = 3, V(0) = 1,~E(0) = 0.3,~I(0) = 2,~R(0) = 0.2$ and the parameter values listed in Table 2.

**Table 2:** Model parameter values.

Parameter	$\Lambda$	$\beta _1$	$\beta _2$	$\sigma$	$\mu$	$\xi$	$\eta$	$\sigma _1$	$\sigma _2$	$\nu$	$\gamma$
Value	1	0.42	0.2	0.2	0.2	0.1	0.2	0.1	0.3	0.1	0.7

**Fig. 4:**
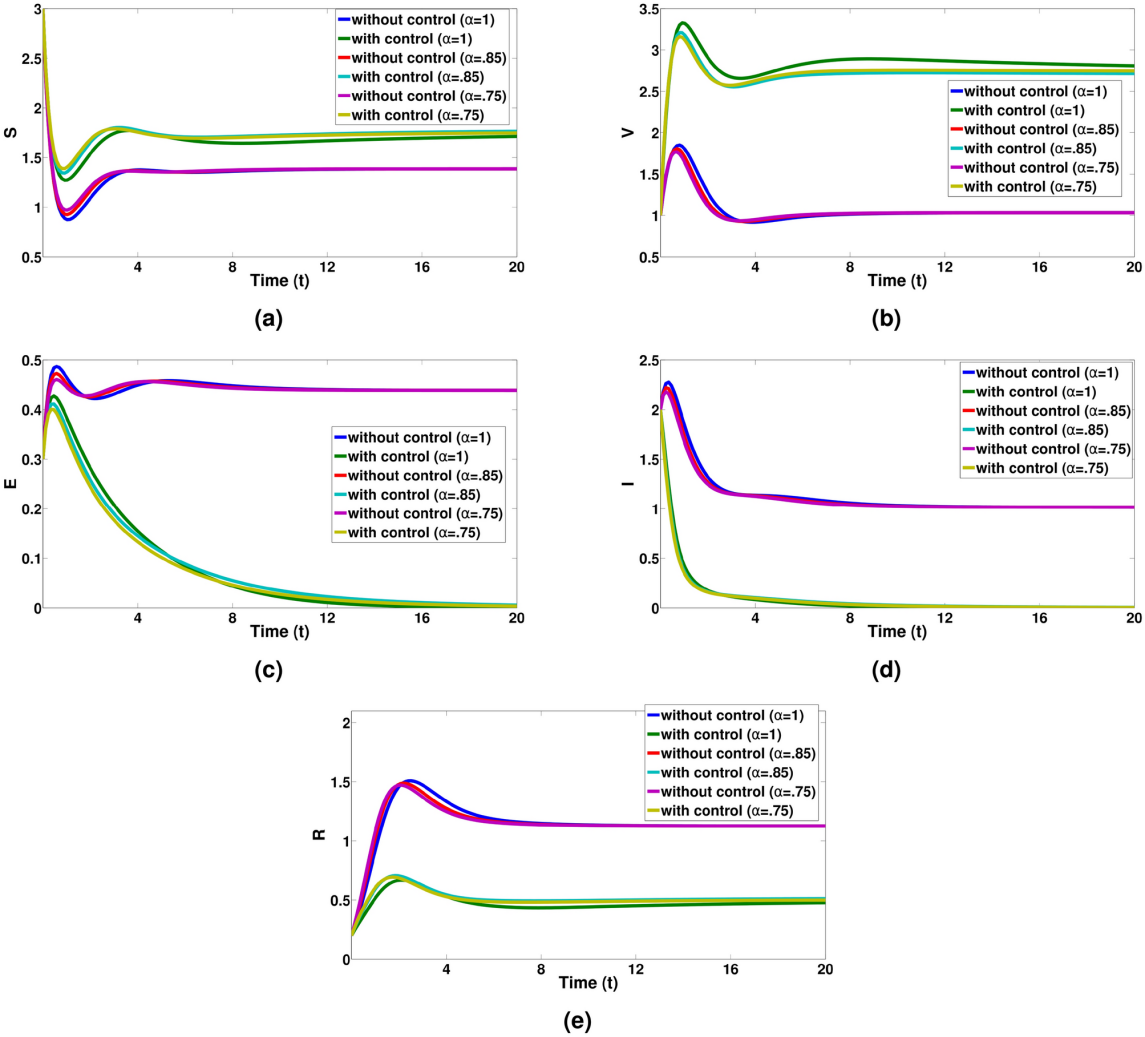
Graphical representation for S, V, E, I and R compartments with and without optimal controls for a integer and non-integer values of $\alpha = 0.75 \& 0.85.$.

**Fig. 5:**
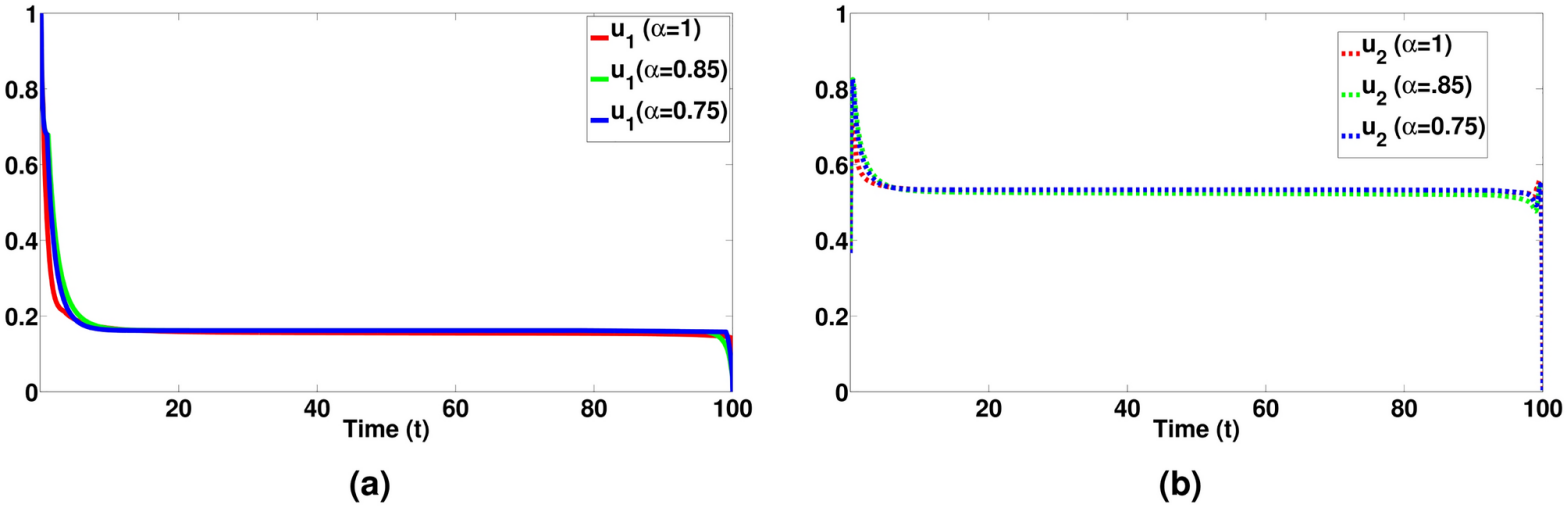
Optimal control trajectory of $u_1 \& u_2$ with integer and non-integer values of $\alpha = 0.75 \& 0.85$.

The numerical simulations are presented in the Fig. 4. In Fig. 4a–e, it is evident that the implementation of control measures leads to a greater increase in the number of susceptible pigs, vaccinated pigs, Exposed pigs, Infected pigs and recovered pigs compared to the scenario without any control measures. The profile of the control variables $u_1$ and $u_2$ are depicts in Fig. 5.

The following is the algorithm used for the numerical simulations to obtain the optimal solution for the proposed system:

#### Algorithm

Step 1: Fix the model parameters and set h, $t_{0}=0$, T and $N = \frac{T}{h}$.
Step 2: Initialize the state variable g(t).
Step 3: Set the $\alpha$ value.
Step 4: Consider the initial condition $g_{0}$. For each time step n = 0, 1, 2,…, N compute the next value of $g_{n+1}$.
Step 5: Perform the Runge kutta method for the proposed state system to obtain the solution without control.
Step 6: Initialize the co-state variables.
Step 7: Perform the state system with control parameters in a forward time loop.
Step 8: Perform the co-state system in the Runge-kutta method over a time backward loop.
Step 9: Modify the control variable by the optimility condition.
Step 10: Calculate the tolerance of error. Iterate until the error is less than prescribed value
Step 11: If loop breaks, repeat from the step 3 for different $\alpha .$
Step 12: Plot the output.
Step 13: End.

Figure 4a reveals that, under control measures, the susceptible pig population is elevated in comparison to the situation where no control measures are in place. The findings presented in Fig. 4b show that when implementing the control inputs $u_1$ and $u_2$ are put into action, the number of vaccinated pigs population is higher rate compared to the situation where no control measures are in respective places. The outcomes shown in Fig. 4c and d strongly indicate the impact of control measures on the populations of exposed and infected pigs. Implementing control parameters results in a substantial decrease in the populations of exposed and infected pigs. Consequently, this reduction in the population of exposed and infected pigs is associated with an increase in the population of recovered pigs as shown in Fig. 4e . Finally from Fig. 4a–e, we can see a notable decline in the populations of exposed and infected pigs when control strategies are put into effect, and by the end of the control period, there is a corresponding increase in the populations of vaccinated and recovered pigs. Therefore, optimal control proves its effectiveness in reducing the populations of exposed and infected pigs within the desired time frame. In Fig. 5, we can observe the control strategies represented by $u_1$ and $u_2$. As shown in Fig. 5a, there is an initial implementation of tightening biosecurity measures, aimed at isolating vaccinated pigs to prevent infections and minimize viral transmission within the farm. These measures are gradually relaxed as the intervention progresses towards its conclusion. In Fig. 5b, we see a different strategy where an effective disinfectant is initially administered to all pigs, with the possibility of increasing its usage over time as the number of recovered pigs rises. Timely vaccination efforts facilitate the transition of pigs from the susceptible (S) compartment to the vaccinated (V) compartment, thereby reducing the exposed and infected populations. If $u_1(t)$ is applied consistently and effectively, the susceptible population will decrease rapidly, while the vaccinated population will grow, slowing the spread of the disease. Similarly, disinfection efforts governed by $u_2(t)$ help to reduce virus transmission in the environment, lowering the numbers of exposed (E) and infected (I) pigs over time. Proper sanitation significantly curbs the spread of ASF, ensuring that fewer susceptible animals become exposed or infected.

## Conclusions

In this research, we have developed a Caputo fractional order mathematical model to describe the transmission behaviours of the ASFV within the SVEIR framework. Our study encompasses an analysis of solution positivity and boundedness of the system. we computed the basic reproductive number ${\mathscr {R}}_{0}$ for our SVEIR model. Following that, we derive conditions that ensure the DFE point exhibits both local and global asymptotic stability. The sensitivity study was performed on this model by using ${\mathscr {R}}_{0}$. Furthermore, we introduced a FOCP and derived the necessary optimality conditions using the Pontryagin maximum principle. To gain insights into the system behaviour, we conducted numerical simulations employing the FRK4M method, and we solved the resulting optimality system numerically by developing the FBSM with FRK4M. Through the implementation of control variables such as enforced biosafety measures and the use of effective disinfectants, we can effectively manage and curb the spread of the African swine fever virus. Also, we conclude from the simulation results, the incorporation of vaccination compartments into our model represents a novel approach compared to existing ASFV models^1,44–49^. The important features of our work are as follows:

- The proposed fractional-order model presents notable benefits by accounting for memory effects, enhancing flexibility and precision, capturing non-local dynamics, and offering improved tools for control and optimization.
- Our findings demonstrate its remarkable effectiveness, highlighting it as the optimal strategy for disease eradication.
- The graphical results demonstrate that the model yields greater adaptability and richer outcomes. These attributes make fractional-order models better suited for modeling complex, real-world systems compared to traditional integer-order models.
- This approach enhances the accuracy of predictions regarding disease spread and control measures, allowing for more effective prevention and intervention strategies.

### Limitations of the current work

- Future studies employ actual ASFV case data to optimize parameter values in the model. Moreover, we aim to expand the model to incorporate a more complex and realistic network framework.
- A stochastic modeling approach that accounts for uncertainty in the dynamics of ASFV could also be explored.
- Time delays are a widely recognized feature of epidemic models. In our future research, we intend to broaden this study by including time delays.

## Data Availability

The datasets used and/or analysed during the current study available from the corresponding author on reasonable request.
